# What is the allowed volume threshold for chest tube removal after lobectomy: A randomized controlled trial

**DOI:** 10.1016/j.amsu.2019.05.011

**Published:** 2019-05-30

**Authors:** Nozomu Motono, Shun Iwai, Aika Funasaki, Atsushi Sekimura, Katsuo Usuda, Hidetaka Uramoto

**Affiliations:** Department of Thoracic Surgery, Kanazawa Medical University, 1-1 Daigaku, Uchinada, Ishikawa, 920-0293, Japan

**Keywords:** Pleural effusion, Volume threshold, Pulmonary resection

## Abstract

**Introduction:**

The management of chest tubes and the volume threshold for chest tube removal after pulmonary resection remain controversial. Several studies have reported the volume threshold for chest tube removal following pulmonary resection to range from 200 to 450 mL/24 h.

**Methods:**

A prospective randomized single-blind clinical study was performed with data collected from patients who had undergone lobectomy and lymph node dissection at our hospital between June 2014 and April 2018. The patients were randomly assigned to the High group (removal of chest tube when drainage was <450 mL/24 h) or Low group (removal of chest tube when drainage was <200 mL/24 h) at postoperative day (POD) 2. The primary end point was drainage time. The secondary end point were complications and rate of thoracentesis.

**Results:**

Seventy patients met the inclusion criteria and were randomized, with 35 patients assigned to the High group and 35 patients to the Low group. The average duration of chest tube placement was 2.05 days in the High group and 2.31 days in the Low group. The duration of chest tube placement in the High group was significantly shorter than that in the Low group (p = 0.02). There were no major postoperative complications in either group. Thoracentesis was not necessary in either group.

**Conclusion:**

Pleural effusion of 450 mL/day is tolerable as the volume threshold for the removal of a chest tube after pulmonary resection.

## Highlights

• Volume threshold for chest tube removal after pulmonary resection is controversial.
• Patients were randomly assigned to the High group (450 mL) or Low group (200 mL).
• The duration of chest tube placement in the High group was significantly shorter.
• There were no major postoperative complications in either group.
• Thoracentesis was not necessary in either group.

## 1 Introduction

Chest tube placement following pulmonary resection is a common modality. The timing of the removal of the chest tubes is often empirically established, and surgeons apply different rules for chest tube management. Several studies have reported the volume threshold for chest tube removal following pulmonary resection to range from 200 to 450 mL/24 h [[1], [2], [3], [4], [5], [6], [7]]. The aim of this study was to evaluate the efficacy and safety of the early removal of the chest tube following pulmonary resection.

## 2 Methods

### 2.1 Study design

A prospective randomized single-blind clinical study was performed with data collected from patients who had undergone lobectomy and lymph node dissection at our hospital between June 2014 and April 2018. This study included the patients who underwent more than lobectomy and mediastinal lymphadenectomy, without bleeding, chylothorax, air leakage, or thoracic infection at 2 days after surgery. Patients were excluded if they were younger than 19 years of age and older than 85 years of age, underwent lobectomy with chest wall resection, or underwent pneumonectomy. This study was conducted in accordance with the amended Declaration of Helsinki. The institutional review boards of our hospital approved the protocol (the approval number: R235), and written informed consent was obtained from all patients. This study has been reported in line with the CONSORT criteria and cite the paper above.

### 2.2 Randomization of patients

**Fig. 1:**
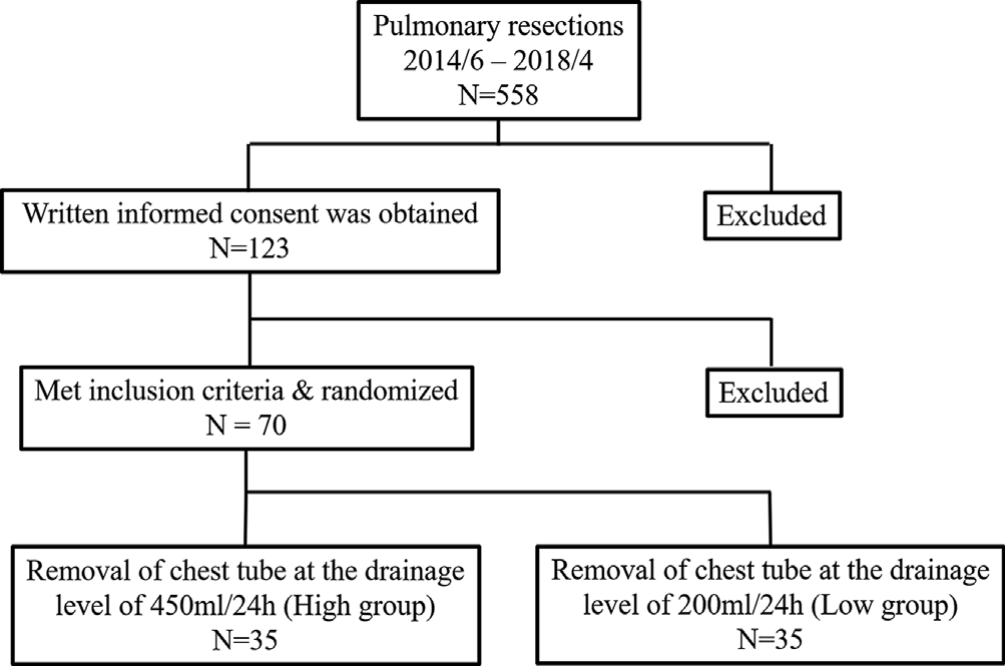
Flow diagram of the removal of the chest tube in the present randomized trial.

The patients were blinded and randomly assigned to the High group (removal of chest tube when drainage was <450 mL/24 h) or the Low group (removal of chest tube when drainage was <200 mL/24 h) at postoperative day (POD) 2 by numbered container method. Fig. 1 shows the flow diagram of the study. One chest tube was inserted and positioned into the anterior apical chest after pulmonary resection. The type of chest tube used in this study was a 20-Fr soft polyvinyl chloride tube. The digital drainage system, Thopaz™ (Medela Healthcare, Zug, Switzerland) was used in this study. The chest tubes were subjected to continuous suction (10 cmH_2_O) until their removal. The tubes were removed when there was no air leakage or densely bloody and chylous pleural effusion.

### 2.3 End points

The drainage time (from the day of the operation until the chest tube was removed) and amount of drainage from the chest tube were recorded. Postoperative complications were also recorded. A physical examination was performed every day, and chest X-rays were taken every day until the removal of chest tube. The presence of fluid in the pleural space (identified by X-ray, a physical examination, and patient symptoms) was determined. After discharge, the patients were instructed to have routine follow-up at our hospital: Physical examination and chest X-ray were repeated at 7, and 30 days. The primary end point was drainage time. The secondary end point were complications and rate of thoracentesis.

### 2.4 Statistical analysis

Sample size was calculated using the optimal effectiveness test sample size calculation formulas. Type I error was 0.05 with a two-tailed test and type II error was 0.20. After the calculation, the sample size of each group was decided 35 patients. Fisher's exact test or the χ^2^ test was used to assess categorical variables. Wilcoxon's test was used to assess continuous variables. All statistical analyses were carried out with the JMP software program (Version 13.2; SAS Institute, Inc., Cary, NC, USA). A P-value of <0.05 was considered to be statistically significant.

## 3 Results

### 3.1 Patient characteristics

Between June 2014 and April 2018, 558 patients underwent pulmonary resection for lung cancer. Of these, 123 patients provided their written informed consent. Eight patients who underwent surgery less extensive than lobectomy, lobectomy with chest wall resection, or pneumonectomy were excluded. Seventy-five patients who had air leakage or densely bloody or chylous pleural effusion at POD 2 were excluded. Ultimately, 70 patients met the inclusion criteria and were randomized as 35 patients to the High group (removal of chest tube when drainage was <450 mL/24 h) and 35 patients to the Low group (removal of chest tube when drainage was <200 mL/24 h) at POD 2. Patients’ preoperative data are shown in Table 1. The proportion of patients with a comorbidity (i.e. hypertension or diabetes mellitus) was significantly higher in the High group than in the Low group (High group vs. Low group = 71% vs. 42%, p = 0.01).

**Table 1:** Preoperative data.

Variable	High group (35)	Low group (35)	P Value
Sex			0.47
Male	18	15	
Female	17	20	
Age,y (median, range)	68.4 (58–78)	69.3 (45–84)	0.64
Smoking index (median, range)	0 (0–2520)	100 (0–3290)	0.96
Comorbidity	25 (71%)	15 (42%)	0.01
BSA, m^2^ (median, range)	1.63 (1.25–1.96)	1.64 (1.27–1.95)	0.50
%VC (median, range)	99.6 (77–127.6)	105.9 (81.9–133.3)	0.16
FEV_1.0_% (median, range)	76.4 (32.8–112.1)	75.3 (55.6–109.8)	0.67
CEA, ng/ml (median, range)	4.1 (0.8–15.3)	3.2 (0.9–70)	0.62
TP_pre_, g/dL (median, range)	7.2 (6.2–8.3)	7.1 (6.2–7.9)	0.50
Alb_pre_, g/dL (median, range)	4.2 (3.6–4.9)	4.3 (3.5–5)	0.33

BSA, body surface area; %VC, percent vital capacity; FEV_1.0_%, percent predicted forced expiratory volume in 1 s; CEA, carcinoembryonic antigen; TP_pre_, preoperative level of serum total protein; Alb_pre_, preoperative level of serum albumin.

Perioperative data are shown in Table 2. Sixty-nine patients (98%) underwent lobectomy, and 1 patient (2%) underwent bilobectomy. Operative procedures were performed by video-assisted thoracic surgery (VATS) in 65 patients (93%) and open thoracotomy in 5 patients (7%). There were no significant differences between the two groups in terms of the resected lung lobes and areas of lymph node dissection. Furthermore, the wound length, operation time, and amount of bleeding were not significantly different between the two groups.

**Table 2:** Perioperative data.

Variables	High group (35)	Low group (35)	P Value
Procedure			0.75
RUL	15	12	
RML	2	2	
RMLL	1	0	
RLL	5	9	
LUL	5	6	
LLL	7	6	
Lymph node dissection			0.99
2a-1	22	22	
2a-2	13	13	
Approach			0.74
C-VATS	17	15	
Hybrid VATS	15	18	
Thoracotomy	3	2	
Retractor	9 (25%)	11 (31%)	0.59
Wound length, mm (median, range)	60 (40–120)	60 (30–200)	0.44
Operation time, min (median, range)	175 (108–414)	166 (86–456)	0.22
Amount of bleeding, mL (median, range)	60 (10–435)	40 (10–370)	0.18
Amount of intraoperative infusion, mL (median, range)	1100 (650–1880)	1050 (650–1870)	0.70

RUL, right upper lobectomy; RML, right middle lobectomy; RMLL, right middle and lower lobectomy; RLL, right lower lobectomy; LUL, left upper lobectomy; LLL, left lower lobectomy; VATS, video-assisted thoracic surgery.

### 3.2 Safety of this study

Postoperative data is shown in Table 3. The data of blood examinations were not significantly different between the two groups. The median volume of drainage between POD 0 and POD 1 (D0) was 200 mL in the High group and 175 mL in the Low group, respectively. D0 was not significantly different between the 2 groups (p = 0.77). The median volume of drainage between POD1 and POD 2 (D1) was 250 mL in the High group and 180 mL in the Low group, with a significant difference between the two groups (p = 002). In contrast, the median volume of drainage between POD 0 to POD 2 (D0+1) was 450 mL in the High group and 400 mL in the Low group, with no significant difference between the 2 groups (p = 0.31). The average duration of chest tube placement was 2.05 days in the High group and 2.31 days in the Low group. The duration of chest tube placement in the High group was significantly shorter than that in the Low group (p = 0.02). There were no major postoperative complications in 68 patients (97.2%). Atrial fibrillation developed in 1 patient (1.4%) in each group. Thoracentesis was not necessary in either group.

**Table 3:** Postoperative data.

Variables	High group (35)	Low group (35)	P Value
WBC_max_,/μL (median, range)	11210 (6930–19580)	11530 (7180–18660)	0.61
LDH_max_, U/L (median, range)	213 (161–274)	211 (164–335)	0.56
CRP_max_, mg/dL (median, range)	6.97 (2.27–26.26)	7.82 (3.05–22.3)	0.89
BNP_max_, pg/dL (median, range)	37.4 (10.8–355.9)	49.6 (19.9–157.3)	0.04
TP_min_, g/dL (median, range)	5.8 (5.3–7)	5.7 (5.1–6.8)	0.18
Alb_min_, g/dL (median, range)	3.2 (2.7–3.9)	3.1 (2.5–3.8)	0.32
ΔTP, g/dL (median, range)	1.4 (0.5–2.2)	1.3 (0.6–2)	0.88
ΔAlb, g/dL (median, range)	1 (0.4–1.7)	1.1 (0.6–1.8)	0.08
D0, mL (median:range)	200 (30–400)	175 (50–500)	0.77
D1, mL (median:range)	250 (10–525)	180 (60–750)	0.02
D0+1, mL (median:range)	450 (210–800)	400 (110–975)	0.31
Drainage time, days (mean ± SD)	2.05 ± 0.53	2.31 ± 0.58	0.02
Thoracentesis	0	0	0.99
Morbidity	1, AF	1, AF	0.99

WBC_max_, maximum count of white blood cell; LDH_max_, maximum level of lactate dehydrogenase; CRP_max_, maximum level of C-reactive protein; BNP_max_, maximum level of brain natriuretic peptide; TP_min_, minimum level of serum total protein; Alb_min_, minimum level of serum albumin, ΔTP, gap between TP_pre_ and TP_min_; ΔAlb, gap between Alb_pre_ and Alb_min_; D0, volume of drainage between POD 0 and POD 1; D1, volume of drainage between POD 1 and POD 2; D0+1, volume of drainage between POD 0 and POD 2; AF, atrial fibrillation.

### 3.3 Factors affecting the volume of pleural effusion

Factors affecting the volume of pleural effusion after pulmonary resection were analyzed. D0+1 was significantly correlated with the age, preoperative serum albumin (Alb_pre_), operation time, and amount of intraoperative infusion (Table 4). There was a positive correlation between D0+1 and the age (correlation coefficient; r = 0.24, p = 0.04), operation time (r = 0.35, p < 0.01), and amount of intraoperative infusion (r = 0.24, p = 0.03). In contrast, the Alb_pre_ (r = −0.29, p = 0.01) was negatively correlated with D0+1. Furthermore, the presence of a comorbidity significantly influenced the D0+1 (presence of comorbidity vs. absence of comorbidity = 450 mL vs. 377.5 mL, p = 0.01; data are shown in Table 5).

**Table 4:** Correlation between volume of drainage and patient data.

Variables	Correlation coefficient	P value
Age	0.24	0.04
Smoking index	−0.02	0.82
BSA	0.08	0.50
%VC	−0.04	0.72
FEV_1.0_%	0.12	0.30
CEA	0.12	0.39
TP_pre_	−0.10	0.39
Alb_pre_	−0.29	0.01
Operation time	0.35	<0.01
Wound length	0.01	0.95
Amount of bleeding	0.12	0.30
Amount of intraoperative infusion	0.24	0.03
WBC_max_	0.02	0.88
LDH_max_	−0.06	0.59
CRP_max_	0.08	0.47
BNP_max_	0.05	0.64
TP_min_	−0.07	0.52
Alb_min_	−0.21	0.06
ΔTP	−0.04	0.71
ΔAlb	−0.12	0.29

BSA, body surface area; %VC, percent vital capacity; FEV_1.0_%, percent predicted forced expiratory volume in 1 s; CEA, carcinoembryonic antigen; TP_pre_, preoperative level of serum total protein; Alb_pre_, preoperative level of serum albumin; WBC_max_, maximum count of white blood cell; LDH_max_, maximum level of lactate dehydrogenase; CRP_max_, maximum level of C-reactive protein; BNP_max_, maximum level of brain natriuretic peptide; TP_min_, minimum level of serum total protein; Alb_min_, minimum level of serum albumin, ΔTP, gap between TP_pre_ and TP_min_; ΔAlb, gap between Alb_pre_ and Alb_min_.

**Table 5:** Relationship between volume of drainage and patient data.

Variable	D0+1	P Value
Sex		0.47
Male	450	
Female	420	
Comorbidity		0.01
Absent	377.5	
Present	450	
Procedure	1.63	0.59
RUL	410	
RML	400	
RMLL	675	
RLL	450	
LUL	410	
LLL	500	
Lymph node dissection		0.09
2a-1	405	
2a-2	450	
Approach		0.46
C-VATS	450	
Hybrid VATS	395	
Thoracotomy	475	
Retractor		0.41
Absent	440	
Present	400	

D0+1, volume of drainage between POD 0 and POD 2; RUL, right upper lobectomy; RML, right middle lobectomy; RMLL, right middle and lower lobectomy; RLL, right lower lobectomy; LUL, left upper lobectomy; LLL, left lower lobectomy; VATS, video-assisted thoracic surgery.

## 4 Discussion

The management of chest tubes and the volume threshold for chest tube removal after pulmonary resection remain controversial [[1], [2], [3], [4], [5], [6], [7]]. The decision to remove the tube is based on the lack of air leakage, densely bloody and chylous pleural effusion, and a decrease in the volume. The volume thresholds reportedly range from 200 to 450 mL/day. The present study revealed the safety of removing the chest tube after pulmonary resection when the drainage level was ≤450 mL/24 h. Pleural fluid is filtered and mostly reabsorbed at the parietal lymphatics [8]. The maximum pleural lymph flow is believed to be 700 mL/day. An increase in the pleural filtration rate beyond the maximum pleural lymph flow results in pleural effusion. Therefore, pleural effusion of 450 mL/day is deemed tolerable as the volume threshold for the removal of the chest tube after pulmonary resection.

Previous studies have reported distinct pleural fluid characteristics, depending on the time of onset of effusion in the postoperative period [[9], [10], [11], [12], [13]]. In thoracic surgery, postoperative pleural effusion is a neutrophilic exudate in the early phase and subsequently lymphocytic. In general, the postoperative pleural effusion in the early phase is considered to be due to damage of the pleura, whereas the pleural effusion in the late phase is considered to be more likely due to an immune-inflammatory process related to operative stress. However, the approach of operation (VAT vs thoracotomy), use of a retractor, and wound length did not correlate with the volume of postoperative pleural effusion in the present study. The volume of postoperative pleural effusion might have no relation to the damage of the pleura.

The age, Alb_pre_, operation time, and amount of intraoperative infusion influenced pleural effusion in the present study. A low level of serum albumin usually causes low oncotic pressure and might thereby increase the transudative pleural effusion. Age and Alb_pre_ might cause low oncotic pressure. The operation time significantly correlated with the amount of intraoperative infusion (r = 0.80, p < 0.01). Because longer operation times result in greater intraoperative infusion, a longer operation time might be associated with a lower oncotic pressure. The minimum level of serum albumin (Alb_min_) in the postoperative data tended to correlate negatively with the volume of pleural effusion. Alb_min_ might influence the increased transudative pleural effusion for the same reason.

The presence of a comorbidity significantly influenced the postoperative pleural effusion in the present study. A previous study demonstrated that a history of heart failure, more advanced and diffuse arteriosclerosis, and atrial fibrillation were significantly more prevalent among patients with pleural effusion [11]. Sixteen patients with hypertension, 13 patients with diabetes mellitus, and 4 patients with angina pectoris were included in the present study. Although no patients had heart failure, patients with arteriosclerosis may still have been included in the present study.

The present study has several limitations. Because a pleural fluid analysis was not performed, the pleural effusion could not be differentiated as exudative or transudative. In addition, we were unable to include all patients who underwent pulmonary resection during this period, so the number of patients was small.

## 5 Conclusions

We reported that a pleural effusion of 450 mL/day is tolerable as the volume threshold for the removal of a chest tube after pulmonary resection. Furthermore, the age, Alb, and volume of intraoperative infusion might influence the pleural effusion due to oncotic pressure.

## Ethical approval

The institutional review boards of Kanazawa Medical University approved the protocol (the approval number: R235).

## Sources of funding

None.

## Author contribution

Conception and design: N.M.

Administrative support: N.M.

Provision of study materials or patients: N.M.

Collection and assembly of data: N.M., S.I., A.F., A.S., and K.U.

Data analysis and interpretation: N.M. and H.U.

Manuscript writing: All authors.

Final approval of manuscript: All authors.

## Conflicts of interest

None.

## Trial registry number

Japanese clinical trial registry UMIN000013930.

https://upload.umin.ac.jp/cgi-open-bin/ctr/ctr_view.cgi?recptno=R000015260.

## Guarantor

Nozomu Motono and Hidetaka Uramoto.

## Provenance and peer review

Not commissioned, externally peer reviewed.
